# Incidence of near-death experiences in patients surviving a prolonged critical illness and their long-term impact: a prospective observational study

**DOI:** 10.1186/s13054-023-04348-2

**Published:** 2023-02-27

**Authors:** Anne-Françoise Rousseau, Laurence Dams, Quentin Massart, Laila Choquer, Héléna Cassol, Steven Laureys, Benoit Misset, Nadia Dardenne, Olivia Gosseries, Charlotte Martial

**Affiliations:** 1 Department of Intensive Care and Burn Centre, University Hospital of Liège, University of Liège, Liège, Belgium; 2 Department of Anaesthesiology, University Hospital of Liège, University of Liège, Liège, Belgium; 3 Coma Science Group, GIGA-Consciousness, University of Liège, Avenue de L’hôpital, 11, 4000 Liège, Belgium; 4 Centre du Cerveau², University Hospital of Liège, Liège, Belgium; 5 Joint International Research Unit On Consciousness, CERVO Brain Research Centre, CIUSS, University Laval, Québec, Canada; 6 University and Hospital Biostatistics Centre (B-STAT), University of Liège, Liège, Belgium; 7 Sensation and Perception Research Group, GIGA-Consciousness, University of Liège, Liege, Belgium

**Keywords:** Near-death experience, Dissociation, Prospective study, Intensive care

## Abstract

**Background:**

So far, the few prospective studies on near-death experience (NDE) were carried out only in intensive care unit (ICU) patients with homogeneous aetiologies, such as cardiac arrest or trauma survivors. The aims of this 1-year prospective and monocentric study were to investigate the incidence of NDE in ICU survivors (all aetiologies) as well as factors that may affect its frequency, and to assess quality of life up to 1 year after enrolment.

**Methods:**

We enrolled adults with a prolonged ICU stay (> 7 days). During the first 7 days after discharge, all eligible patients were assessed in a face-to-face interview for NDE using the Greyson NDE scale, dissociative experiences using the Dissociative Experience Scale, and spirituality beliefs using the WHOQOL-SRPB. Medical parameters were prospectively collected. At 1-year after inclusion, patients were contacted by phone to measure quality of life using the EuroQol five-dimensional questionnaire.

**Results:**

Out of the 126 included patients, 19 patients (15%) reported having experienced a NDE as identified by the Greyson NDE scale (i.e. cut-off score ≥ 7/32). In univariate analyses, mechanical ventilation, sedation, analgesia, reason for admission, primary organ dysfunction, dissociative and spiritual propensities were associated with the emergence of NDE. In multivariate logistic regression analysis, only the dissociative and spiritual propensity strongly predicted the emergence of NDE. One year later (n = 61), the NDE was not significantly associated with quality of life.

**Conclusions:**

The recall of NDE is not so rare in the ICU. In our cohort, cognitive and spiritual factors outweighed medical parameters as predictors of the emergence of NDE.

*Trial registration* This trial was registered in Clinicaltrials.gov in February 2020 (NCT04279171).

**Supplementary Information:**

The online version contains supplementary material available at 10.1186/s13054-023-04348-2.

## Background

Unexpected intense subjective experiences can be experienced at the approach of death or in critical medical situations. These so-called near-death experiences (NDEs) are episodes of disconnected consciousness (i.e. mental content in which concurrent experience of the external world is either absent or very limited) [1] that can be identified by prototypical features (e.g. out-of-body experience) [2]. With advances in critical care medicine over the past few decades, more patients are surviving an intensive care unit (ICU) stay [3] and report such NDEs. Patients in ICU may face potentially physical stressors, such as inflammation, high catecholamine levels, independently of the primary organ failure triggering ICU admission [4]. These are all potential inducers of NDE [5]. Next to these (neuro)physiological factors, some cognitive processes have also been proposed to trigger NDE, such as the tendency for dissociation [6]. Nevertheless, so far, no prospective study has been conducted to investigate the incidence of NDEs across all aetiologies ICU survivors.

The primary aim of this monocentric study was to describe the incidence of NDEs among patients who survived a prolonged ICU stay. The secondary aims were to search for medical and cognitive factors that may affect the frequency of NDEs, to assess the NDE memory at one month, and to assess the 1-year impact of NDEs on quality of life.

## Methods

Detailed method (including statistical analyses) is provided in the Additional file 1. Between July 2019 and March 2020, all patients who were discharged from the 5 ICUs of our hospital after a prolonged stay (i.e. > 7 days) were screened. All enrolled patients were screened for delirium within the first week after ICU discharge, using the *Confusion Assessment Method* (CAM). Patients detected with confusion or delirium were reassessed during their ward stay, and further included in case of confusion or delirium resolution. At hospital, during the first 7 days after ICU discharge, all eligible patients were assessed in a face-to-face interview for potential NDE using the *Greyson NDE scale* [7]. The *Dissociative Experience Scale* (DES) and the *World Health Organization Quality of Life-Spirituality, Religiousness and Personal Beliefs* (WHOQOL-SRPB) were then administrated to, respectively, assess frequency of both pathological and non-pathological dissociative experiences and spirituality, religiousness and personal beliefs. Based on their response to the Greyson NDE scale (i.e. cut-off score ≥ 7/32 for a NDE), the cohort was then divided into two groups: the *NDE group* and the *non-NDE group*.

At one month following the interview, patients from the NDE group were contacted by phone to measure the phenomenological characteristics of memories using the *Memory Characteristics Questionnaire* (MCQ). We also asked them if they had already experienced a similar subjective experience before their ICU admission.

Finally, patients from both groups were contacted by phone one year after inclusion, to assess their memory using the Greyson NDE scale and their health-related quality of life (HRQoL) using the *EuroQol five-dimensional questionnaire*. Patients were also questioned about their feeling during the ICU stay of living a life-threatening situation (yes/no answer).

Demographic data, medical history and data related to the ICU stay were prospectively extracted from the medical charts.

This study was conducted according to the guidelines of the Declaration of Helsinki, after approval by the Ethics Committee of the Medicine Faculty of the University of Liège (2019/104) and registration on ClinicalTrials.gov (NCT04279171). Informed consent was obtained from all the patients.

## Results

### NDE incidence

One hundred twenty-six participants were included in the study and interviewed 5 (3–7) days after ICU discharge (Additional file 1: Figure S1 for enrolment flowchart). Out of this sample, 19 patients (15%) reported having experienced a NDE as identified by the Greyson NDE scale (NDE group). All other 107 patients were included in the non-NDE group.

### Patient characteristics

The two groups did not differ with regard to sociodemographic characteristics and medical history (Additional file 1: Table S1).

### ICU stay

The ICU characteristics in the two groups are detailed in Table 1. The two groups differed in terms of reason for admission, primary organ dysfunction, mechanical ventilation, sedation, and analgesia. However, there was no statistical difference between the two groups in terms of recorded physiological parameters during the ICU stay (lowest arterial systolic blood pressure, partial arterial oxygen and carbon dioxide pressure, oxygen saturation, arterial pH or body temperature, and highest partial arterial carbon dioxide pressure or body temperature).

**Table 1:** Intensive Care Unit (ICU) stay characteristics in NDE (n = 19) and non-NDE groups (n = 107)

Variables		NDE group n = 19 (%)	Non-NDE group n = 107 (%)	Statistic	*p*-value
Reason for admission *No. of patients (%)*	Surgical	11 (58)	33 (31)	Chi^2^ test = 5.20	0.023*
	Medical	8 (42)	74 (69)		
Primary organ dysfunction *No. of patients (%)*	Cardio-vascular	9 (47)	35 (33)	Fisher’s exact test = 15.84	0.024*
	Respiratory	2 (10)	43 (40)		
	Digestive	4 (21)	3 (3)		
	Renal	0 (0)	3 (3)		
	Neurologic	1 (5)	6 (6)		
	Metabolic	1 (5)	3 (3)		
	Trauma	2 (10)	12 (11)		
	Other	0 (0)	2 (2)		
ICU length of stay *Median in days (Q1–Q3)*		10 (8–16)	12 (9–18)	Kruskal–Wallis = 0.51	0.48
SAPS II *Median (Q1–Q3)*		33 (23–41)	34 (25–40)	Kruskal–Wallis = 0.070	0.79
Mechanical ventilation *No. of patients (%)*		16 (84)	62 (58)	Fisher’s exact test = 0.039	0.039*
Duration of mechanical ventilation *Median in days (Q1–Q3)*		4.5 (2–9)	5 (2–10)	Kruskal–Wallis = 0.46	0.50
Noradrenaline *No. of patients (%)*		12 (63)	63 (59)	Chi^2^ test = 0.73	0.12
Sedation *No. of patients (%)*		17 (89)	67 (63)	Chi^2^ test = 5.24	0.022*
Type of sedation *No. of patients (%)*	Midazolam	3 (17.6)	10 (14.9)	Fisher’s exact test = 0.077	0.72
	Propofol	16 (94.1)	62 (92.5)	Fisher’s exact test = 0.051	1.00
	Clonidine	4 (23.5)	11 (16.4)	Fisher’s exact test = 0.47	0.49
	Dexmedetomidine	2 (11.8)	10 (14.9)	Fisher’s exact test = 0.11	1.00
	Sevoflurane	1 (5.9)	1 (1.5)	Fisher’s exact test = 1.12	0.37
Analgesia *No. of patients (%)*		16 (84)	65 (61)	Chi^2^ test = 3.87	0.049*
Primary analgesic *No. of patients (%)*	Sufentanyl	14 (87)	59 (91)	Chi^2^ test = 0.15	0.65
	Remifentanil	2 (12)	6 (9)		
Co-analgesic *No. of patients (%)*	None	13 (81)	49 (75)	Chi^2^ test = 1.30	0.86
	Remifentanil	0 (0)	3 (5)		
	Piritramide	1 (6)	2 (3)		
	Ketamine	2 (12)	11 (17)		
Neuromuscular blocking agents *No. of patients (%)*		2 (10)	8 (7)	Chi^2^ test = 0.21	0.65
Nitric oxide *No. of patients (%)*		0 (0)	1 (5)	Fisher’s exact test = 5.68	0.15
Renal replacement therapy *No. of patients (%)*		4 (21)	13 (12)	Fisher’s exact test = 1.096	0.29
Extracorporeal membrane oxygenation *No. of patients (%)*		0 (0)	4 (4)	Chi^2^ test = 0.73	1.00
Cardiac arrest *No. of patients (%)*		1 (5)	5 (5)	Fisher’s exact test = 0.012	1.00

Q1–Q3 = first and third quartiles; SD = standard deviation; T = temperature; SAPS II = simplified acute physiology score; SpO2 = peripheral oxygen saturation; PaCO2 = partial pressure of arterial carbon dioxide; PaO2 = partial pressure of arterial oxygen

*p < 0.05

### Questionnaires

We found highly significant differences between the two groups for all the questionnaires (Additional file 1: Table S2). The final model of multivariate binary logistic regression analysis revealed the DES and the WHOQOL-SRPB as the strongest predictors for the emergence of NDE, with an odds ratios (OR) = 1.13 (95% confidence interval [CI] 1.04–1.24; p = 0.005) and with an OR = 1.48 (95% CI 1.20–1.82; *p* = 0.0002), respectively (Additional file 1: Table S3).

Analysis of the DES-T revealed that only one patient from the NDE group reached the cut-off of 15 with a score of 18.75, placing his score in the pathological dissociative class.

The Greyson total scores were positively correlated with the SRPB total score and its five subscores (Additional file 1: Table S4).

### Follow-up at 1 month

In the NDE group, 10 of the 19 patients were followed at 1 month after enrolment (Additional file 1: Figure S1). Patients’ scores on the MCQ revealed a high amount of qualitative phenomenological memory characteristics (Additional file 1: Table S5).

### Follow-up at 1 year

Out of the 126 included patients, 50 and 11 patients in the non-NDE and NDE groups, respectively, were contacted by phone at 1 year after inclusion. No significant differences were observed between both groups for all questionnaires, except for the Greyson NDE scale (Additional file 1: Table S6).

The Greyson total score did not change 1 year later, in the whole sample of patients we were able to contact by phone at 1 year after inclusion (*p* = 0.46) and in each group: NDE group (*p* = 0.086) and non-NDE group (*p* = 0.84). The Greyson total score evolution between the first and second administration was similar in the two groups (*p* = 0.065).

## Discussion

This prospective study revealed a NDE incidence of 15% in patients discharged from the ICU after a prolonged stay, no matter what critical illness they survived. This incidence is consistent with the 10–23% incidence observed in cardiac arrest survivors [8–12], but higher than the studies reporting a 4–8% prevalence estimation among the general population [13, 14].

**Fig. 1:**
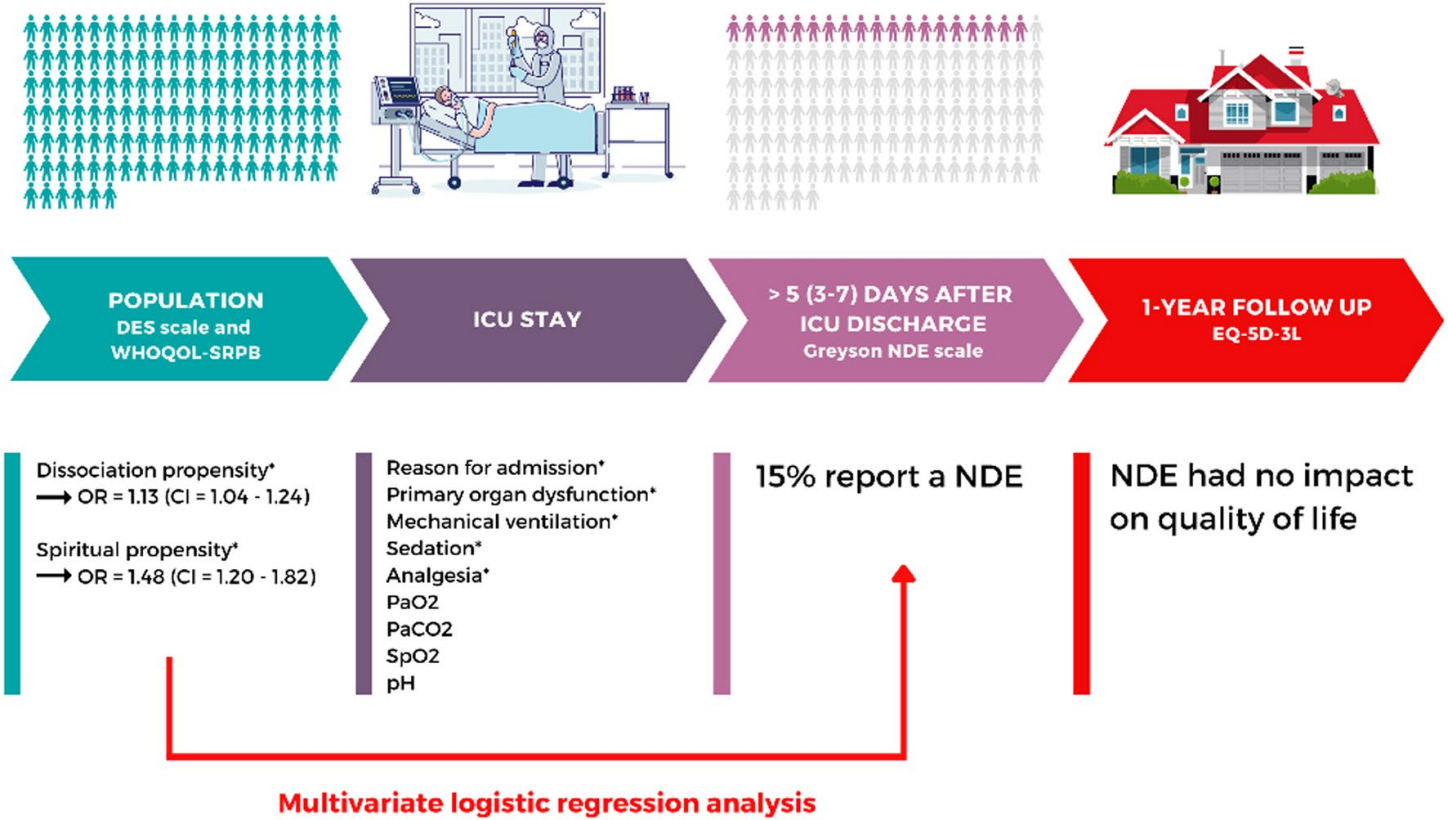
Summary of the results of this 1-year prospective study. *p < 0.05; NDE = near-death experience; ICU = intensive care unit; DES = Dissociative Experience Scale; WHOQOL-SRPB = World Health Organization Quality of Life-Spirituality, Religiousness and Personal Beliefs; EQ-5D-3L = EuroQol five-dimensional questionnaire; PaO2 = partial pressure of arterial oxygen; PaCO2 = partial pressure of arterial carbon dioxide; SpO2 = peripheral oxygen saturation; OR = odds ratios; CI = confidence interval

In univariate analyses, we observed that seven variables were associated with the emergence of NDE; however, only a higher frequency of dissociative symptoms and a greater spiritual and personal well-being were the strongest predictors for the recall of NDE using multivariate analysis (Fig. 1). It is then reasonable to hypothesize that a propensity to dissociative states and to spiritual beliefs and practices make people more likely to report NDEs when exposed to certain physiological conditions. This corroborates Greyson’s retrospective study [6] showing more (non-pathological) dissociative symptoms in NDE population. As suggested by Noyes and Slymen [15], dissociation would offer a less distressing “reality” to people facing with a potential danger. This is also consistent with the hypothesis suggesting that NDEs may have a specific biological benefit when facing life-threatening situations [16]. Spiritual well-being is an important coping resource as well [17].

Yet, it is not thought possible to explain NDEs only in terms of psychological processes. Absence of proof is not proof of absence. The current literature converges to say that several neurophysiological mechanisms may provoke the occurrence of NDEs [1, 5]. Obviously, it was impossible to objectively determine when exactly during the ICU stay were NDEs experienced and if NDEs were related to the initial event leading to ICU admission or to subsequent events during the ICU stay. This may explain that none of the recorded physiological parameters have been found as a risk factor for NDE.

One year later, the ICU-related NDE was not significantly associated with HRQoL measured. Similarly, the perception of a life-threatening situation was not impacted a posteriori by the previous occurrence of NDE. Finally, for all our patients, the Greyson total score did not change 1 year later.

NDEs are typically reported as transforming [18] and may be associated with negative emotions [19]. This is why we consider clinically meaningful to interview patients about any potential memory upon awakening.

Some limitations need to be acknowledged. First, the number of patients who experienced a NDE were limited. This could have limited the analysis of NDE risk factors, as well as making false negatives more likely. Yet, patients whose experience did not reach the validated cut-off score of 7/32 for a typical NDE were not categorized as NDE experiencers, and some patients may have denied or forgotten to have lived a NDE. Second, the large number of comparisons increases the probability of type 1 errors; however, the level of significance of individual comparisons for the questionnaires was well below the 0.05 threshold, thereby reducing the likelihood of false positives. Finally, delirium during the ICU stay was not routinely assessed. It is still unknown if delirium could be a risk factor for NDE.

In conclusion, we observed a NDE incidence of 15% and that cognitive and spiritual outweighed medical parameters as predictors of the emergence of NDE. Further studies are needed to confirm these findings in larger cohorts or in survivors of a shorter ICI stay.

## Supplementary Information

**Additional file 1**. Detailed method (including statistical analyses).

## Data Availability

The datasets generated during and/or analysed during the current study are available from the corresponding author on reasonable request.

## Abbreviations

NDE: Near-death experience
ICU: Intensive care unit
DES: Dissociative Experience Scale
WHOQOL-SRPB: World Health Organization Quality of Life-Spirituality, Religiousness and Personal Beliefs
MCQ: Memory Characteristics Questionnaire
HRQoL: Health-related quality of life
SD: Standard deviation
OR: Odds ratios
CI: Confidence interval
IQR: Interquartile range
T: Temperature
SAPS II: Simplified acute physiology score
SpO2: Peripheral oxygen saturation
PaCO2: Partial pressure of arterial carbon dioxide
PaO2: Partial pressure of arterial oxygen
