# Supplementary material for: Incidence of near-death experiences in patients surviving a prolonged critical illness and their long-term impact: a prospective observational study
Source: Crit Care. 2023 Feb 27;27:76. doi: 10.1186/s13054-023-04348-2 (PMC9969623; doi:10.1186/s13054-023-04348-2)
Supplement: Supplementary file 1 — Additional file 1. Detailed method (including statistical analyses). [file 13054_2023_4348_MOESM1_ESM.docx]

**Supplemental Materials**

**Supplemental Material 1. Method description**

Study population and procedure

Exclusion criteria were: <18 years old, non-French speaking, refusal to participate, confusion or delirium detected by the Confusion Assessment Method (CAM) instrument, and early hospital discharge to home, inpatient rehabilitation or another hospital.

Questionnaires

* The Confusion Assessment Method (CAM) instrument [1] is a four-feature standardized and validated tool that enables non-psychiatrically trained clinicians to identify and recognize confusion and delirium quickly in both clinical and research settings. Patients detected with confusion or delirium (i.e., presence of features 1 and 2 and either 3 or 4) were further excluded from the study.

* The French version (back-translated method) of the Greyson NDE scale [2] was used to identify potential NDE. This 16-item multiple-choice validated scale is useful in differentiating NDEs from organic brain syndromes, nonspecific stress responses and any other kind of subjective experiences. Patients who reached the validated cut-off score (i.e., ≥7/32) were considered NDE experiencers (i.e., NDE group).

* The French version (back-translated method) of the 28-item Dissociative Experience Scale (DES) [3] permits to assess frequency and types of both pathological and non-pathological dissociative experiences. Each item describes a specific daily life experience, reflecting different forms of dissociative symptoms (depersonalization and derealization, absorption/imaginative involvement, dissociative amnesia). Respondents are asked to report the percentage of the time they had the experience described, on a scale ranging from 0% (never) to 100% (always). A subject's score ranges from 0 to 100 for each item of the scale, and the result is obtained by calculating the average of the total scores. Scores ≥25 indicate a tendency to dissociation trait. A DES-T score can also be calculated to identify pathological dissociation (cut-off score of 15) [4], which corresponds to the average of eight items (items 3, 5, 7, 8, 12, 13, 22, and 27).

* The World Health Organization Quality Of Life-Spirituality, Religiousness and Personal Beliefs (WHOQOL-SRPB) [5] assesses religious, spiritual, and personal beliefs (French version) [6]. This questionnaire is composed of 32 items regrouped in eight subscales (four items each): hope and optimism, meaning of life, awe, inner peace, wholeness and integration, spiritual strength, connectedness to a spiritual being or force, and faith. Each item is graded by using a 5-point Likert scale (ranging from 1 “not at all or very unsatisfied” to 5 “extremely/completely agree or very satisfied”). Each of the 4 items contributes equally to the subscale score. Mean scores are then calculated: all the items in the respective subscale are added and divided by four. Each subscale score is taken to contribute equally to the total score. Total score ranges from 0 to 20 with higher scores suggesting greater spiritual well-being (considered here as an expression of spiritual maturity).

* The modified (French) version [7] of the Memory Characteristics Questionnaire (MCQ) [8] is built to analyze phenomenological characteristics of real and imagined memories. This questionnaire encompasses 16 rating scales assessing feeling of re-experiencing, visual details, other sensory details, location, time, coherence, verbal component, emotion while remembering, belief that the event is real, one’s own actions, words and thoughts, visual perspective, emotional valence, personal importance, and reactivation frequency. A MCQ total score was derived summing all the 16 items (each on a 1–7 point Likert scale) and referred to as the amount of memory characteristics (i.e., higher total scores reflect greater amount of memory characteristics).

***** The EuroQol five-dimensional questionnaire (EQ-5D-3L) [9] evaluates health-related quality of life. This tool comprises two sections. The first section is a five-question descriptive component which explores five dimensions (mobility, self-care, usual activities, pain/discomfort and anxiety/depression). Each question has three possible answers, rated from 1 to 3: no problems, some problems and extreme problems. The second section is a visual analogue scale (EQ VAS; 0 “worst imaginable health state” to 100 “best imaginable health state”) about their current health state.

Statistical analyses

Since it was an observational study with no previous existing data in such category of patients, no sample size was calculated.

Statistical analysis was performed using SAS (version 9.4 for Windows, SAS Institute, Cary, NC, USA) and R (version 4.0.2 for Windows) software. The normality of the quantitative variables was tested with the Shapiro–Wilk test (p<0.05). Results were expressed as mean and standard deviation (SD) or as medians with interquartile range (Q1-Q3) for asymmetric distribution. Qualitative variables were described using count and percent. Pearson’s χ² (or Fisher exact test if appropriate) was used to assess the homogeneity of the distributions between the two groups. T-tests or non-parametric Kruskal–Wallis tests were used to determine the statistical differences among groups for continuous variables. Variables that differed significantly between NDE and non-NDE groups in the univariate analysis (p<0.05) were included in a multivariate binary logistic regression analysis to identify variables that remained significantly associated with the emergence of NDE. Same multivariate regression with a Stepwise procedure was also computed. Using the Firth method, we estimated the odds ratios (OR) with 95% confidence interval (95% CI). The Area Under the Receiver Operating Characteristic Curve (AUC) with 95% confidence interval was also reported for the original data and after cross validation. Values could range from 0 to 1 where AUC of 0.5 suggests no discrimination, values from 0.7 to 0.8 are considered acceptable, from 0.8 to 0.9 as excellent, and more than 0.9 as outstanding [10]. In the NDE group, we also calculated Spearman rank-order correlations between the Greyson total score and respectively the DES total score, the WHOQOL-SRPB total score and its sub-scores.

Wilcoxon signed-rank tests were performed to assess potential differences in the Greyson NDE score at hospital time and one year later in the whole sample of patients and within each group. Mann-Whitney U test was used to compare the Greyson NDE total score evolution from hospital time to the one-year phone call between the two groups.

The results were considered significant at the level of α=5% (p<0.05).

References:

1. Inouye SK, van Dyck CH, Alessi CA, Balkin S, Siegal AP, Horwitz RI. Clarifying confusion: the confusion assessment method. A new method for detection of delirium. Ann Intern Med. 1990;113(12):941-8

2. Greyson B. The near-death experience scale. Construction, reliability, and validity. J Nerv Ment Dis. 1983;171(6):369-75

3. Bernstein EM, Putnam FW. Development, reliability, and validity of a dissociation scale. J Nerv Ment Dis. 1986;174(12):727-35

4. Waller N, Putnam FW, Carlson E. Types of dissociation and dissociative types: A taxonomic analysis of dissociative experiences. Psychol Methods. 1996;1:300-21

5. Group WS. A cross-cultural study of spirituality, religion, and personal beliefs as components of quality of life. Soc Sci Med. 2006;62(6):1486-97

6. Mandhouj O, Etter JF, Courvoisier D, Aubin HJ. French-language version of the World Health Organization quality of life spirituality, religiousness and personal beliefs instrument. Health Qual Life Outcomes. 2012;10:39

7. D'Argembeau A, Van der Linden M. Remembering pride and shame: self-enhancement and the phenomenology of autobiographical memory. Memory. 2008;16(5):538-47

8. Johnson MK, Foley MA, Suengas AG, Raye CL. Phenomenal characteristics of memories for perceived and imagined autobiographical events. J Exp Psychol Gen. 1988;117(4):371-6

9. Rabin R, de Charro F. EQ-5D: a measure of health status from the EuroQol Group. Ann Med. 2001;33(5):337-43

10. Hosmer Jr, D.W., Lemeshow, S., Sturdivant, R.X.: Applied logistic regression. 2013; John Wiley & Sons

**Supplemental Figure 1.** Enrolment flowchart.


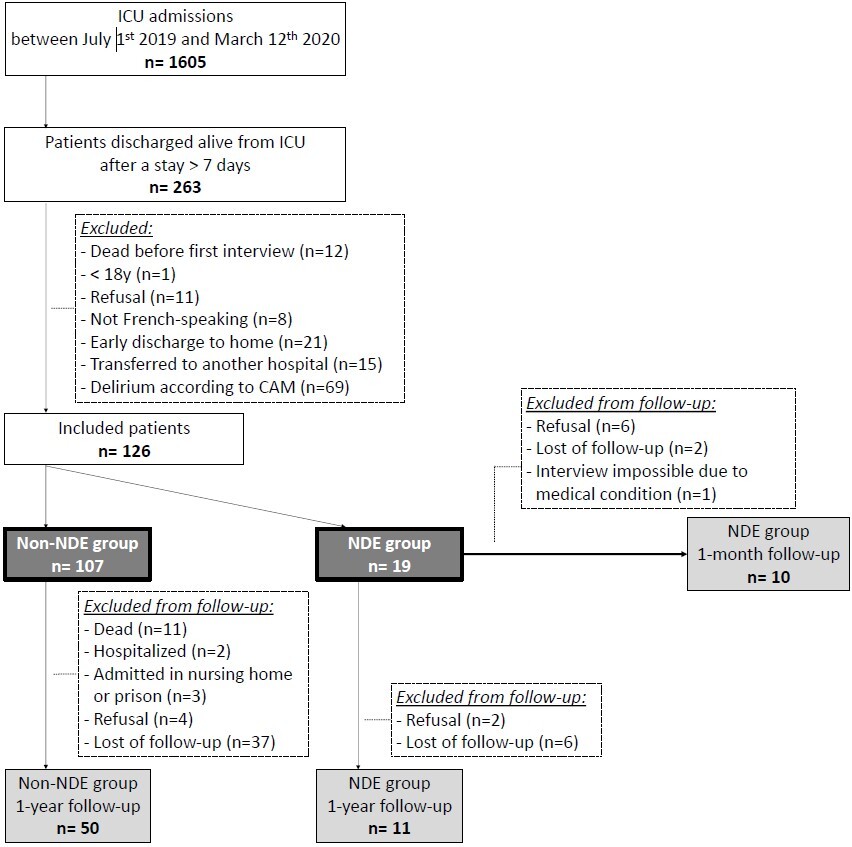


**Supplemental Table 1. Sociodemographic characteristics and medical history of both NDE and non-NDE groups.**

| **Variables** | | **NDE group**  (n=19) | **Non-NDE group**  (n=107) | **Statistic** | **p-value** |
| --- | --- | --- | --- | --- | --- |
| Sex (male)  *No. of patients (%)* | | 10  (53) | 64  (60) | Chi² test = 0.34 | 0.56 |
| Age  *Median in years (Q1-Q3)* | | 57  (46-67) | 64  (56-70) | Kruskal-Wallis = 20.84 | 0.14 |
| Weight  *Median in kg (Q1-Q3)* | | 71  (61-83) | 70  (60-82) | Kruskal-Wallis = 0.011 | 0.92 |
| Size  *Mean in cm (SD)* | | 169  (12) | 170  (9) | T-test = 0.41 | 0.68 |
| BMI  *Median in kg/m² (Q1-Q3)* | | 25  (21-28) | 24  (21-28) | Kruskal-Wallis = 0.12 | 0.73 |
| Addiction (alcohol, tobacco, drug)  *No. of patients (%)* | | 12  (63) | 49  (46) | Chi² test = 1.95 | 0.16 |
| Education level  *No. of patients (%)* | Primary school | 0 | 13  (12) | Fisher’s exact test = 5.54 | 0.12 |
|  | High school | 13  (68) | 77  (72) |  |  |
|  | Bachelor | 4  (21) | 8  (7) |  |  |
|  | Master | 2  (10) | 9  (8) |  |  |
| Culture  *No. of patients (%)* | Occidental | 19  (100) | 106  (99) | Fisher’s exact test = 0.18 | 1.00 |
|  | African | 0  (0) | 1  (1) |  |  |
| Religion  *No. of patients (%)* | Agnostic | 1  (5) | 1  (1) | Fisher’s exact test = 5.69 | 0.17 |
|  | Atheist | 4  (21) | 37  (35) |  |  |
|  | Catholic | 12  (63) | 65  (61) |  |  |
|  | Lay | 0  (0) | 1  (1) |  |  |
|  | Muslim | 1  (5) | 1  (1) |  |  |
|  | Protestant | 1  (5) | 2  (2) |  |  |
| Antecedent  *No. of patients (%)* | Epilepsy | 1  (5) | 6  (6) | Fisher’s exact test = 0.0036 | 1.00 |
|  | Stroke | 1  (5) | 5  (5) | Fisher’s exact test = 0.012 | 1.00 |
|  | Cirrhosis | 2  (10) | 5  (5) | Fisher’s exact test = 1.054 | 0.28 |
|  | Diabetes | 1  (5) | 19  (18) | Fisher’s exact test = 1.89 | 0.30 |
|  | Chronic obstructive pulmonary disease | 5  (26) | 32  (30) | Chi² test = 0.10 | 0.75 |
|  | Heart disease | 5  (26) | 27  (25) | Fisher’s exact test = 0.010 | 1.00 |
|  | Neoplasia | 1  (5) | 21  (20) | Fisher’s exact test = 2.31 | 0.19 |
|  | Psychiatric trouble | 1  (5) | 11  (10) | Fisher’s exact test = 0.47 | 0.69 |
| Other (previous) NDE(s)  *No. of patients (%)* | | 4  (21) | 10  (9) | Fisher’s exact test = 2.24 | 0.22 |

Q1-Q3= first and third quartiles; SD= standard deviation; NDE= near-death experience; BMI= body mass index

**Supplemental Table 2. Total scores and subscores of all questionnaires for the two groups (NDE group: N=19; and non-NDE group: N=107).** The four patients who experienced other NDEs in the past obtained a higher DES total score (median=14; Q1-Q3=12-16) than the ones who did not (median=6; Q1-Q3=4-9) (p=0.027; Kruskal-Wallis=4.86).

| **Questionnaires** | | **NDE group**  (n=19) | **Non-NDE group**  (n=107) | **Kruskal-Wallis**  **Statistic** | **p-value** |
| --- | --- | --- | --- | --- | --- |
| Greyson total score  *Median (Q1-Q3)* | | 10  (8-12) | 2  (1-3) | 49.26 | <0.0001* |
| DES total score  *Median (Q1-Q3)* | | 9  (5-14) | 2  (1-7) | 19.48 | <0.0001* |
| WHOQOL-SRPB | Subscale 1 - Hope & optimism  *Median (Q1-Q3)* | 4  (3-5) | 3  (2-4) | 13.48 | 0.0002* |
|  | Subscale 2 - Meaning of life  *Median (Q1-Q3)* | 4  (3-5) | 3  (2-4) | 19.85 | <0.0001* |
|  | Subscale 3 - Awe  *Median (Q1-Q3)* | 4  (3-5) | 3  (2-4) | 12.58 | 0.0004* |
|  | Subscale 4 - Inner peace  *Median (Q1-Q3)* | 4  (3-5) | 3  (2-4) | 7.44 | 0.0064* |
|  | Subscale 5 - Wholeness & integration  *Median (Q1-Q3)* | 3  (2-4) | 3  (2-3) | 10.84 | 0.0010* |
|  | Subscale 6 - Spiritual strength  *Median (Q1-Q3)* | 4  (3-5) | 2  (1-3) | 16.67 | <0.0001* |
|  | Subscale 7 - Connectedness to a spiritual being or force  *Median (Q1-Q3)* | 3  (3-5) | 2  (1-3) | 16.72 | <0.0001* |
|  | Subscale 8 - Faith  *Median (Q1-Q3)* | 3  (2-4) | 1  (1-3) | 8.55 | 0.0035* |
|  | Total score  *Mean (SD)* | 15  (3) | 10  (3) | 5.58 | <0.0001* |

*p<0.05; Q1-Q3= first and third quartiles; SD= standard deviation

**Supplemental Table 3. Multivariate binary logistic regression models analysing the relationship between significant baseline parameters and NDE occurrence probability**. The Area Under the Receiver Operating Characteristic Curve (AUC) value remained excellent after cross validation (0.85 [0.77 – 0.94]).

| **Model** | **Parameters** | **OR (95% CI)** | **p-value** | **AUC (95% CI)** |
| --- | --- | --- | --- | --- |
| 1^st^ model including all significant parameters in the univariate analysis | Mechanical ventilation  Sedation  Analgesia  DES  SRPB | 6.10 (0.21-176.33)  3.59 (0.22-58.71)  0.14 (0.004-4.77)  1.12 (1.02-1.22)  1.45 (1.28-1.77) | 0.29  0.37  0.28  0.014*  0.0003* | 0.89 (0.82 – 0.95) ^(a)^  0.85 (0.77 – 0.93) ^(b)^ |
| 2^nd^ model – stepwise procedure | DES  SRPB | 1.13 (1.04-1.24)  1.48 (1.20-1.82) | 0.0053*  0.0002* | 0.87 (0.80 – 0.95) ^(a)^  0.85 (0.77 – 0.94) ^(b)^ |

*p<0.05; OR= odds ratios; CI= confidence interval; AUC= area under the receiver operating characteristic curve; (a)= values obtained in the original data; (b)= values obtained after cross validation

**Supplemental Table 4. Spearman rank correlations between the Greyson total with the DES, SRPB total score and subscores (N=19).**

|  | **DES** | **SRPB1** | **SRPB2** | **SRPB3** | **SRPB4** | **SRPB5** | **SRPB6** | **SRPB7** | **SRPB8** | **SRPB total** |
| --- | --- | --- | --- | --- | --- | --- | --- | --- | --- | --- |
| **Greyson total score** | r=0.30  p=0.21 | r=0.36  p=0.13 | r=0.67  p=0.0016* | r=0.63  p=0.0038* | r=0.48  p=0.040* | r=0.55  p=0.014* | r=0.29  p=0.23 | r=0.52  p=0.023* | r=0.20  p=0.40 | r=0.59  p=0.0075* |

*p<0.05

**Supplemental Table 5. NDE group patients’ scores on each of the 16 MCQ items** **(N=10), administered at 1 month after inclusion.**

| **MCQ items** | **Score** |
| --- | --- |
| Feeling of re-experiencing  *Median (IQR)* | 2  (1-7) |
| Visual details  *Median (IQR)* | 7  (6-7) |
| Other sensory details  *Median (IQR)* | 4  (1-5) |
| Location  *Median (IQR)* | 7  (6-7) |
| Time  *Median (IQR)* | 1  (1-3) |
| Coherence  *Median (IQR)* | 5  (4-7) |
| Verbal component  *Median (IQR)* | 5  (1-6) |
| Feeling emotions  *Median (IQR)* | 7  (2-7) |
| Real/imagine  *Median (IQR)* | 7  (7-7) |
| One’s own actions  *Median (IQR)* | 7  (6-7) |
| One’s own words  *Median (IQR)* | 7  (1-7) |
| One’s own thoughts  *Median (IQR)* | 7  (6-7) |
| Visual perspective  *Median (IQR)* | 7  (5-7) |
| Valence  *Median (IQR)* | 6  (2-7) |
| Personal importance  *Median (IQR)* | 7  (3-7) |
| Reactivation frequency  *Median (IQR)* | 6  (3-7) |

IQR=interquartile range

**Supplemental Table 6. Scores of all questionnaires administered at 1 year after inclusion for the two groups.**

| **Questionnaires** | | **NDE group**  (n=11) | **Non-NDE group**  (n=50) | **Statistic** | **p-value** |
| --- | --- | --- | --- | --- | --- |
| Greyson total score  *Median (IQR)* | | 10  (1-14) | 2  (0-4) | Kruskal-Wallis = 6.99 | 0.0082* |
| EQ-5D-3L | Total score  *Median (IQR)* | 7  (7-9) | 7  (6-8) | Kruskal-Wallis = 1.13 | 0.29 |
|  | Visual analogue scale (VAS)  *Median (IQR)* | 70  (50-80) | 65  (50-80) | Kruskal-Wallis = 0.051 | 0.82 |
| Life-threatening situation  *No. of patients (%)* | | 5  (45) | 16  (33) | Chi² test = 0.65 | 0.42 |

*p<0.05; IQR= interquartile range
